# A novel differentiation pathway from CD4^+^ T cells to CD4^−^ T cells for maintaining immune system homeostasis

**DOI:** 10.1038/cddis.2016.83

**Published:** 2016-04-14

**Authors:** X Zhao, G Sun, X Sun, D Tian, K Liu, T Liu, M Cong, H Xu, X Li, W Shi, Y Tian, J Yao, H Guo, D Zhang

**Affiliations:** 1 Experimental and Translational Research Center, Beijing Friendship Hospital, Capital Medical University, Beijing, China; 2 Liver Research Center, Beijing Friendship Hospital, Capital Medical University, Beijing, China; 3 Beijing Key Laboratory of Tolerance Induction and Organ Protection in Transplantation, Beijing, China; 4 Beijing Key Laboratory of Translational Medicine in Liver Cirrhosis, Beijing, China; 5 National Clinical Research Center for Digestive Diseases, Beijing, China

## Abstract

CD4^+^ T lymphocytes are key players in the adaptive immune system and can differentiate into a variety of effector and regulatory T cells. Here, we provide evidence that a novel differentiation pathway of CD4^+^ T cells shifts the balance from a destructive T-cell response to one that favors regulation in an immune-mediated liver injury model. Peripheral CD4^−^CD8^−^NK1.1^−^ double-negative T cells (DNT) was increased following Concanavalin A administration in mice. Adoptive transfer of DNT led to significant protection from hepatocyte necrosis by direct inhibition on the activation of lymphocytes, a process that occurred primarily through the perforin-granzyme B route. These DNT converted from CD4^+^ rather than CD8^+^ T cells, a process primarily regulated by OX40. DNT migrated to the liver through the CXCR3-CXCL9/CXCL10 interaction. In conclusion, we elucidated a novel differentiation pathway from activated CD4^+^ T cells to regulatory DNT cells for maintaining homeostasis of the immune system *in vivo*, and provided key evidence that utilizing this novel differentiation pathway has potential application in the prevention and treatment of autoimmune diseases.

The immune system is maintained at homeostasis under physiological conditions, and immune reactions, when they occur, are well modulated. Without appropriate self-regulation, physiologic processes are at high risk of becoming uncontrolled and even pathologic. Even during pathological processes, the immune system attempts to restore its balance, and activated immune components are appropriately downregulated and then terminated after completion of their mission. This mechanism of self-control is of utmost importance in maintaining immune homeostasis. In contrast to T effector cells, T regulatory cells (Tregs) play a critical role in counteracting an over-activated immune action that occurs in response to exogenous pathogens and self- or allo-antigens. Tregs, therefore, help prevent the perpetuation of diseases and the occurrence of autoimmune diseases^1, 2, 3, 4^ or graft rejection.^5, 6^

Various types of Tregs have been identified, including CD4^+^CD25^+^FOXP3^+^ Tregs, natural killer T cells (NKT), Tr1 CD4^+^ T cells and αβTCR^+^CD3^+^CD4^−^CD8^−^ double-negative T cells (DNT), among others.^7, 8^ Although the latter only comprise a minority (1–5%) of total T cells, it has recently been demonstrated that DNT possess potent immuno-regulatory competence. These cells induce immune tolerance, thereby preventing allograft loss and the onset of autoimmunity.^9, 10, 11^

Immune-mediated liver injury has a critical role in the majority of liver diseases, especially in infectious and autoimmune liver diseases. Concanavalin A (ConA)-induced necro-inflammatory damage of the liver is a well-accepted immunological model of liver injury, which occurs primarily through over-activation of CD4^+^ T cells, Kupffer cells and NKT cells.^12, 13, 14^ It is well known that CD4^+^ T cells can differentiate into Tregs as well as Th1, Th2, Th9, Th22, Tfh and Th17 T cells, depending on the specific cytokine milieu. These various types of T cells develop to exert a variety of different immune functions and to maintain the balance between immune invading and protecting components.^15, 16, 17, 18, 19^

In this study, we revealed a novel differentiation pathway, which converts over-activated CD4^+^ T cells into a subset of regulatory T cells (DNT), which would then serve as an immune defense against ConA-induced liver injury *in vivo,* for maintaining immune system homeostasis.

## Results

### DNT induced by ConA administration in a time- and dose-dependent manner

**Figure 1:**
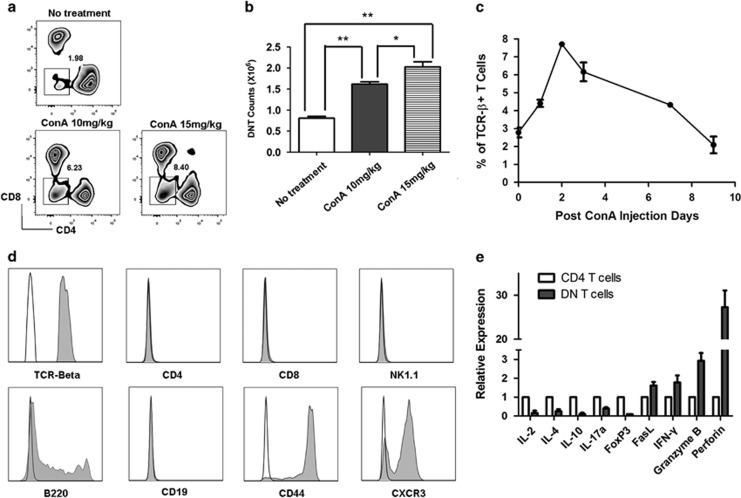
The proportion of CD3^+^CD4^−^CD8^−^ double-negative T cells (DNT) was upregulated following ConA administration in C57BL/6 mice. DNT were significantly induced by ConA stimulation in a dose- ((**a** and **b**), *n=*3 in each group) and time- (**c**) dependent manner (*n=*4). The induced DNT (Gray histograms) highly expressed αβTCR, B220, CD44 and CXCR3, whereas no expression of CD4, CD8, CD19 and NK1.1 was observed. Mouse isotype Ig used as control (white histograms, **d**). Gene expression profile of ConA-induced DNT (**e**). **P<0.01, *P<0.05

Following ConA administration, the T-cell populations in mouse spleens were examined by flow cytometry. As shown in Figure 1a, DNT as a percentage of total CD3^+^TCRβ^+^ T cells increased from the nadir of 1.98 to 6.23% at a ConA dose of 10 mg/kg and to 8.40% at a dose of 15 mg/kg 2 days after treatment (Figure 1a). The absolute counts of DNT in the spleen were also increased significantly (Figure 1b). The dynamic changes in the DNT population were further evaluated. As shown in Figure 1c, the proportion of splenic DNT began to increase from 2.50 to 4.50% on day 1 and reached its maximum level at 8.00% on day 2 after ConA challenge (15 mg/kg). Thereafter, the percentage of DNT began to slowly decline to 6.00% on day 3 and 5.00% on day 7, returning to the original level of 2.00% on day 9. To test whether the increasement of DNT is ConA-specific, we injected a small dose of agonistic CD3 antibody (intravenous injection with 10 μg anti-CD3 antibody, clone: 2c11, purchased from BD Biosciences, San Diego, CA, USA) into wild-type (WT) B6 mice. Results are shown in Supplementary Figures 1a and 1b, the percentage and absolute numbers of DNT in spleens were both increased significantly 48 h after anti-CD3 antibody injection, suggested that the effect on the increasement of DNT *in vivo* is a consequence of T-cell activation, not ConA dependent.

To characterize the DNT after ConA challenge, we examined the expression of cell surface markers and gene profiles. As shown in Figure 1d, DNT were TCRβ^+^ and expressed B220, CD44 and CXCR3, whereas exhibiting no expression of CD4, CD8, CD19 or NK1.1 compared with isotype controls. Real-time PCR was used to analyze the gene expression profile of DNT. As shown in Figure 1e, DNT did not express Foxp3, unlike CD4 T cells, they expressed very low levels of interleukin (IL)-2, IL-4, IL-10 and IL-17a. However, DNT highly expressed Fas ligand (FasL), interferon (IFN)-γ and granzyme B. Perforin, a cytotoxic lymphocyte-related gene, was expressed by DNT at significantly high level.

### ConA-induced DNT are regulatory cells that protect mice from ConA-induced liver injury

**Figure 2:**
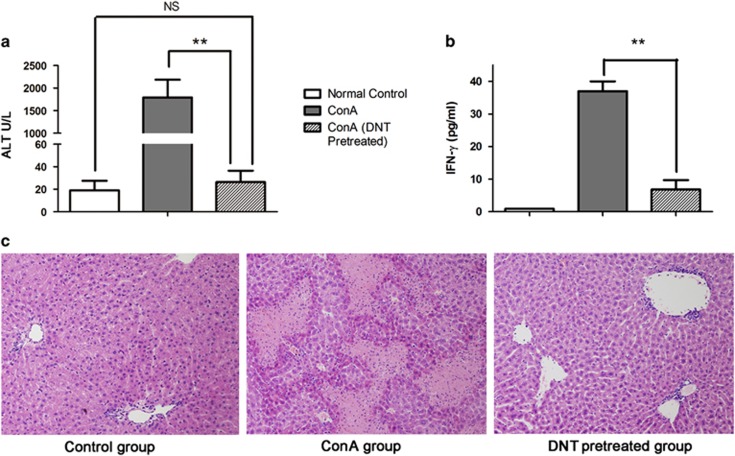
ConA-induced DNT protected the liver from immune-mediated injury. Adoptive transfer of ConA-induced DNT into C57BL/6 mice before ConA administration significantly limited ConA-induced liver injury. Serum aminotransferase (ALT) levels ((**a**), *P<*0.001) and serum IFN-γ levels ((**b**), *P<*0.01, *n=*4 in each group) in the pretreated DNT group were significantly decreased compared with the ConA group (**P<0.01, *P<0.05, NS=no significant difference). The severity of hepatocyte necrosis, shown by pathological analysis, was also significantly reduced in the pretreated DNT group (**c**). Paraffin sections, original magnification x200

To test the functional properties of these increased DNT following ConA challenge, we isolated and adoptively transferred the cells (1 × 10^6^) into WT C57BL/6 mice. In the absence of transferred DNT, serum ALT levels in ConA-treated mice increased significantly up to 2000 U/l 24 h after treatment (1793±393.30 U/l, Figure 2a). Serum IFN-γ, a proinflammatory cytokine, which secreted mainly by activated NKT and T cells and has direct implications for the induction of liver cell injury,^20^ was also significantly increased (37.33±4.23 pg/ml, Figure 2b). This result was in accordance with histopathological changes, such as bridging coagulative necrosis of hepatocytes (Figure 2c). In contrast, the mice that received adoptively transferred DNT before ConA challenge showed significantly lower levels of serum aminotransferase (ALT; 26.35±10.13 U/l, *P<*0.001) and IFN-γ (6.83±2.87 pg/ml, *P*=0.0016). In addition, pathological analysis of these mice revealed no obvious hepatocyte necrosis.

**Figure 3:**
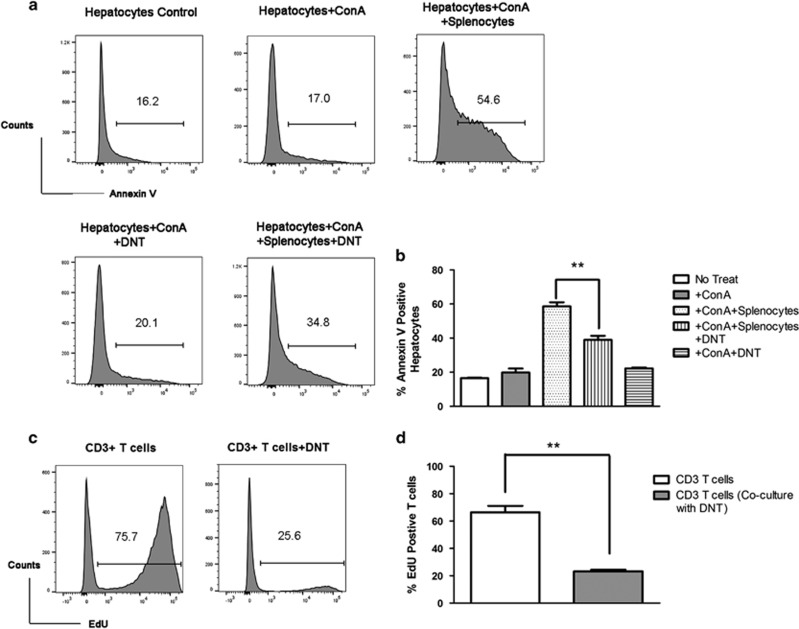
ConA-induced DNT suppressed T-cell proliferation and protected hepatocytes from T cell-mediated injury *in vitro*. *In vitro* hepatocytes cytotoxic assay was assessed by Annexin V staining-positive cells. DNT (from CD45.2 C57BL/6 mice) protected hepatocytes from damage ((**a**), gating on CD45.1^−^CD3^−^ hepatocytes) caused by ConA-activated syngeneic splenocytes. Results are expressed as mean±S.D. of triplicate cultures ((**b**), *P*=0.005) and are representative of three experiments with similar results. Suppressive function of DNT was assessed by EdU incorporation assay (**c** and **d**). Naïve CD3^+^ T cells from CD45.1 C57BL/6 mice were cultured with or without DNT in the presence of anti-CD3 (5 μg/ml) and anti-CD28 (2 μg/ml) for 72 h. Edu was added to the wells for 18 h before harvest. Results are expressed as mean±S.D. of triplicate cultures and are representative of three experiments with similar results. (**P<0.01, *P<0.05)

To further test the immune protection of DNT, *in vitro* cytotoxic assays were performed. After 4 h of ConA stimulation, the apoptosis of hepatocytes was not induced by ConA without co-culturing with splenocytes (The percentage of Annexin V-positive hepatocytes in ConA-treated group and no treat group is 17.00% and 16.20%, respectively.). Annexin V-positive hepatocytes were increased from 17.00 to 54.60% (Figures 3a and b) when cultured with ConA-activated splenocytes. However, ConA-induced DNT protected hepatocytes from immune-mediated injury caused by ConA-activated syngeneic splenocytes (Annexin V-positive hepatocytes were decreased from 54.60 to 34.80%). Interestingly, ConA-induced DNT showed no obvious damage to hepatocytes. *In vitro* suppression assay showed that DNT were capable of inhibiting the proliferation of CD3^+^ T cell (The percentage of 5-ethynyl-2′-deoxyuridine (EdU)-positive T cells decreased from 66.40±4.76% to 23.27±1.68%, Figures 3c and d.). These results suggested that DNT involved in immune protection in ConA-mediated liver injury through direct inhibition on activated lymphocytes. It is notable that the unchallenged mice have a small pool of naïve DNT in peripheral lymph organ. To further test the functional difference of naïve and ConA-induced DNT, we isolated DNT from either ConA-treated or naïve B6 mice, and tested their suppressive function on T-cell proliferation. *In vitro* suppression assay showed that DNT from naïve B6 mice were capable of inhibiting the proliferation of CD3^+^ T cell (The percentage of EdU-positive T cells decreased from 52.00±2.08 to 40.27±2.42%, Supplementary Figures 2A and B.). DNT from ConA-treated mice had more profound suppression on the proliferation of CD3^+^ T cells, the percentage of EdU-positive T cells further decreased to 13.03±1.29% (Supplementary Figures 2A and B). However, the apoptosis rates tested by Annexin V staining did not show significant difference (Supplementary Figure 2C). Which suggests that ConA-induced DNT had more profound regulation on CD3^+^ T cells than Naïve DNT. The regulation of DNT on CD3^+^ T cells were mainly by direct inhibition on activation and proliferation of CD3^+^ T cells in this co-culture system.

### DNT induced by ConA challenge are converted from CD4^+^ rather than CD8^+^ T cells

**Figure 4:**
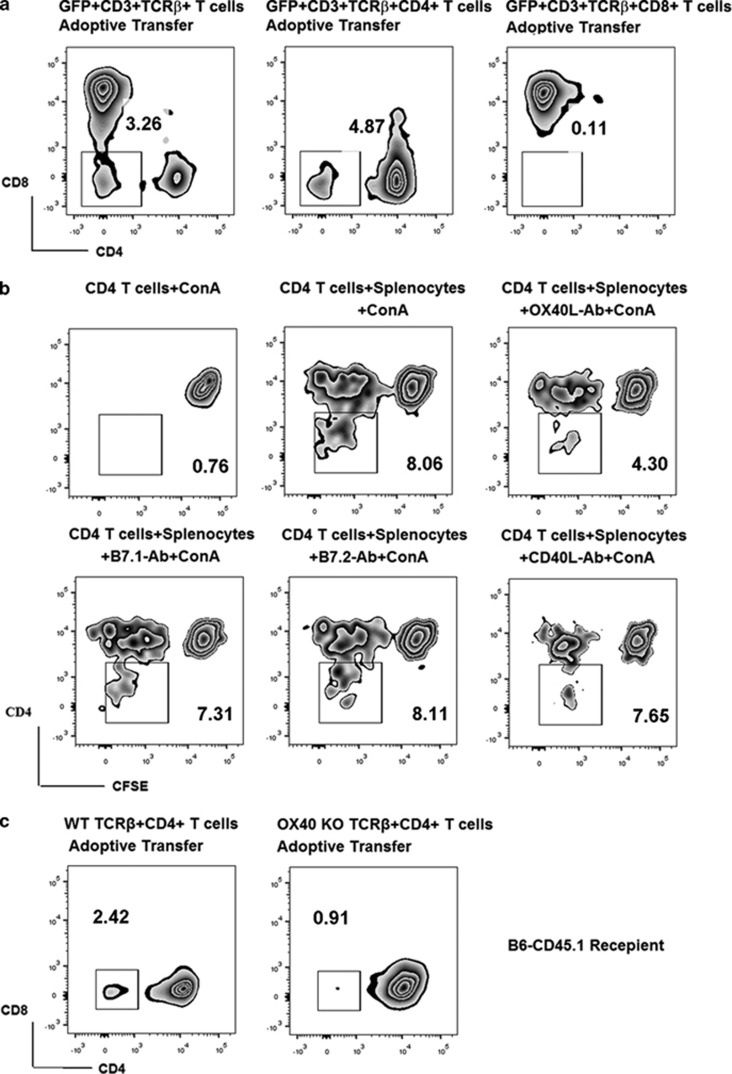
ConA-induced DNT were converted from CD4^+^ rather than CD8^+^ T cells both *in vivo* and *in vitro*, and the conversion of CD4^+^ T cells to DNT was APC-dependent and modulated by the OX40/OX40L interaction. GFP^+^CD4^+^ cells, but not CD8^+^ T cells, were converted into DNT *in vivo* after ConA treatment (**a**). Conversion from CD4^+^ T cells to DNT was observed only in the presence of splenocytes in our *in vitro* co-culture system. This conversion was significantly reduced when the OX40–OX40L interaction was blocked (**b**). Accordingly, conversion from OX40-KO CD4^+^T cells into DNT was significantly reduced *in vivo* (**c**).The results reported are representative of three experiments with similar results

To examine the origin of the increased DNT, highly purified (>98% purity) CD3^**+**^, CD4^+^ or CD8^+^ T cells from GFP^+^C57BL/6 mice were adoptively transferred to WT C57BL/6 mice, followed by ConA injection. As shown in Figure 4a, 48 h after ConA administration, we observed that 3.26% of the total GFP^+^CD3^+^ T cells were DNT in the spleen, from the adoptive transfer group. In the GFP^+^CD4^+^ T-cell adoptive transfer group, DNT reached 4.87% of the total GFP^+^CD3^+^ T cells, whereas almost no (0.11%) GFP^+^DNT appeared in the GFP^+^CD8^+^ T-cell adoptive transfer group.

To further investigate the mechanism of conversion from CD4^+^ T cells to DNT after ConA stimulation, DNT induction tests were performed *in vitro*. CD45.2^+^ CD4^+^ T cells (>98% purity) from WT C57BL/6 mice were cultured alone under ConA stimulation. No CD4^+^ T-cell proliferation or DNT conversion was observed. Next, we co-cultured CD4^+^ T cells with syngeneic splenocytes from CD45.1 C57BL/6 mice under ConA stimulation (5 μg/ml). After several rounds of proliferation, 8.06% of CD45.2^+^ T cells were DNT (Figure 4b). This result suggested that conversion from CD4^+^ T cells to DNT was dependent on antigen-presenting cells (APCs), and that conversion only occurred after several rounds of proliferation. It is important to note that parallel studies were also performed on CD8^+^ T cells, and no conversion from CD8^+^ T cells to DNT occurred (data not shown).

### OX40 plays a key role in modulating the phenotypic differentiation from CD4^+^ T cells to DNT

Because DNT conversion is APC dependent, we investigated the involvement of co-stimulation pathways in this process. B7.1-, B7.2-, CD40L- and OX40L-blocking antibodies were added to the co-culture system. As shown in Figure 4b, only the OX40L-blocking antibody inhibited DNT conversion, dropping the proportion of DNT from 8.06 to 4.30%. These data suggested that the OX40–OX40L interaction plays a very important role in the conversion of CD4^+^ T cells to DNT. To confirm this result *in vivo*, highly purified CD45.2^+^CD4^+^ T cells were isolated from either C57BL/6 WT or C57BL/6 OX40-knockout (KO) mice and then adoptively transferred into CD45.1 C57BL/6 mice. ConA was then administered to the recipient mice. Although 2.42% of WT CD4^+^ T cells converted into DNT, only 0.91% CD4^+^ T cells from OX40-KO mice did so (Figure 4c). Taken together, these results confirmed that DNT convert from CD4^+^ T cells rather than CD8^+^ T cells, not only *in vitro* but also *in vivo*. In addition, OX40 is a key factor in modulating CD4^+^ T-cell conversion to DNT.

### DNT home to the liver to exert a protective function, and the interaction between CXCR3 and its ligands CXCL9/ CXCL10 plays an important role in DNT migration

**Figure 5:**
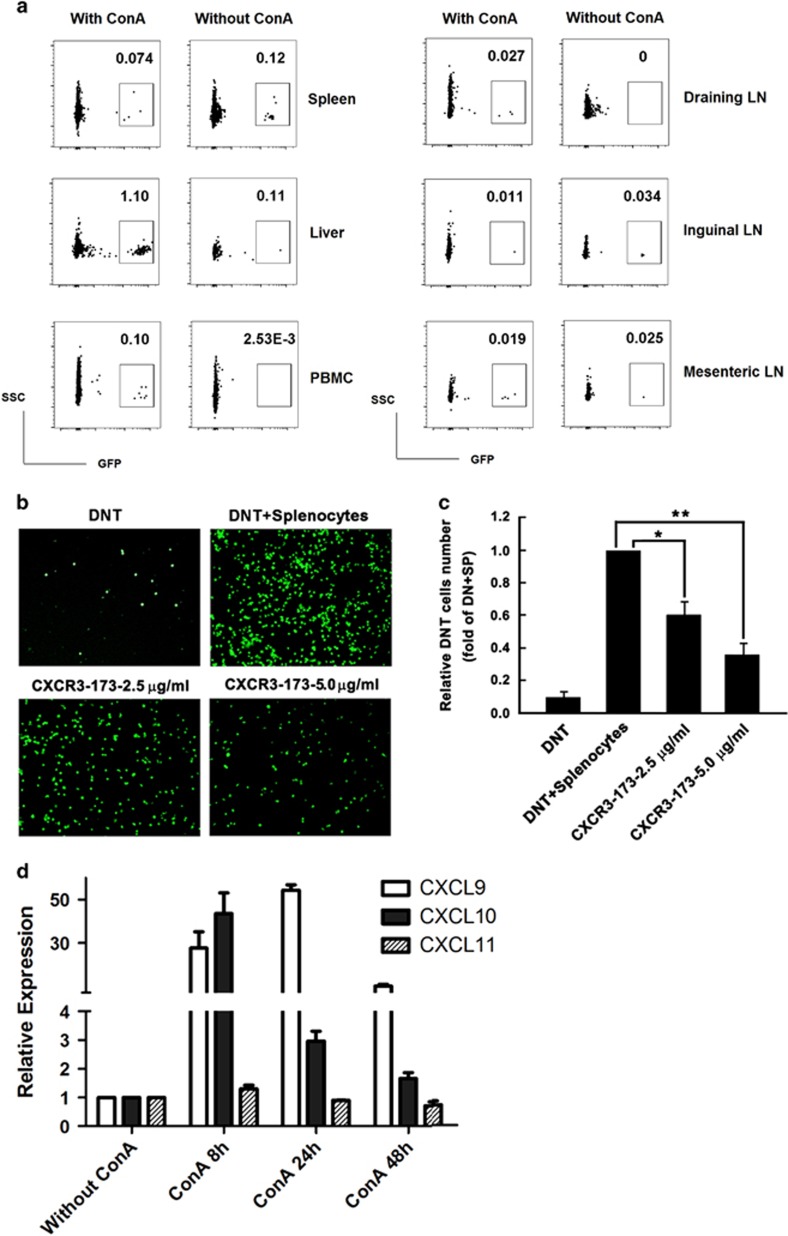
DNT homed to the injured liver via the CXCR3-CXCL9/CXCL10 interaction to exert a protective function. ConA-induced DNT (GFP^+^) migrated to the injured liver after ConA administration (**a**). The migration of GFP^+^DNT was significantly reduced with increasing concentrations of CXCR3-neutralizing mAb tested by *in vitro* migration assay (**b**), and the migration inhibition was dose dependent (**c**). Real-time PCR results showed that mRNA of the CXCR3 ligand CXCL9/10, but not CXCL11, was highly expressed in the injured livers (**d**). The results reported are representative of three experiments with similar results. **P<0.01, *P<0.05

To verify whether the transferred DNT home primarily to the liver to exert their protective function, 1 × 10^6^ GFP^+^ DNT purified from ConA-treated C57BL/6 GFP^+^ mice were adoptively transferred to WT C57BL/6 recipients. These recipient animals were then either treated with ConA or control saline. Without ConA treatment, GFP^+^DNT were randomly distributed to different lymphoid organs, as shown in Figure 5a. With ConA treatment, 10 fold more DNT (percentage of total CD3 T cells) accumulated in the liver than the spleen and in inguinal or mesenteric lymph nodes. Compared with control group, higher proportion of DNT were found in the liver, draining lymph node and peripheral blood mononuclear cell in ConA-treated mice.

It is well known that different chemokine receptors are expressed and play preferential roles in inducing migration of different lymphocyte subpopulations. In this study, we observed that DNT from ConA-induced liver injury expressed high levels of CXCR3 protein (Figure 1d). To confirm that CXCR3 is important for DNT migration, *in vitro* blocking studies were performed. As shown in Figures 5b and c, GFP^+^DNT migration significantly decreased in a dose-dependent manner upon treatment with increasing concentrations of CXCR3-neutralizing monoclonal antibody (mAb). Meanwhile, real-time PCR showed that mRNA of the CXCR3 ligand CXCL9/10, but not CXCL11, was highly expressed in the injured livers (Figure 5d). Taken together, these results suggest that upregulated CXCL9/10 attracted the highly expressed CXCR3 on the surface of DNT, leading to migration of these cells into injured liver where they could exert their protective function.

### Perforin is highly expressed by DNT, modulating the magnitude of the immune response

**Figure 6:**
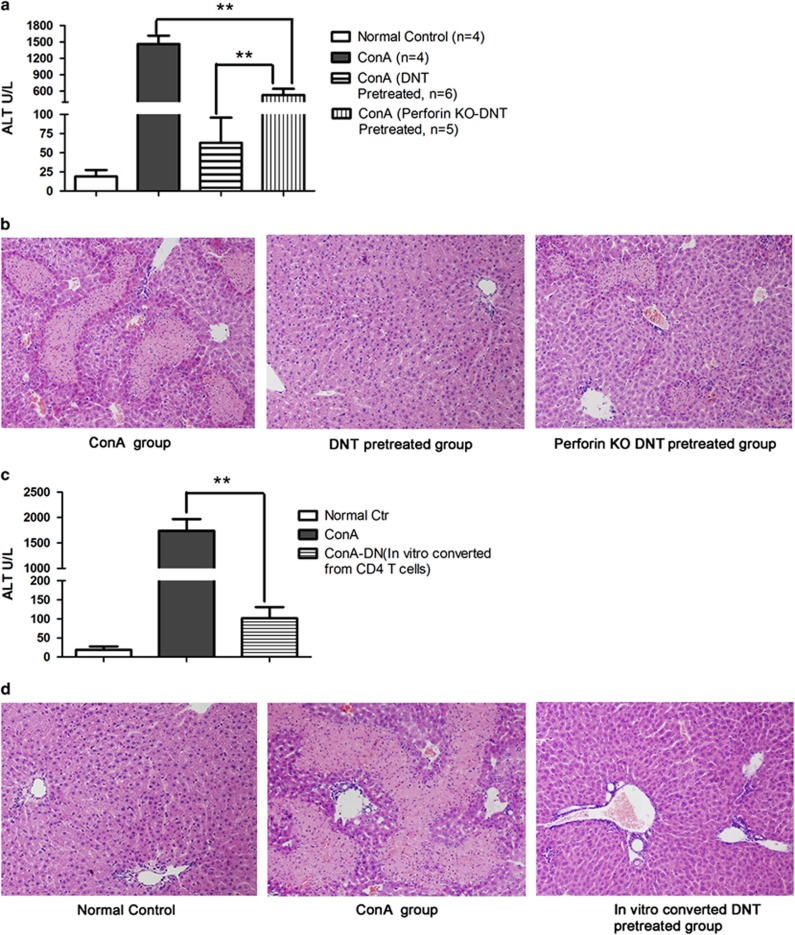
ConA-induced DNT exerted their immune modulation function primarily through the perforin pathway. Adoptive transferring of *ex vivo* converting CD4^+^ T cells to DNT also significantly protected liver from immune-mediated injury. ConA-induced DNT from either WT or perforin-KO animals were transferred into WT C57BL/6 mice, followed by ConA treatment. The protective competence of DNT toward ConA-induced liver damage was significantly reduced in the perforin-KO DNT-preventive group, which manifested as significantly higher ALT levels (**a**) and obvious hepatocyte necrosis compared with the pretreated WT DNT (**b**). Paraffin sections, original magnification: × 200. *Ex vivo* CD4^+^ T cells converted DNT were transferred into WT C57BL/6 mice, followed by ConA treatment. *Ex vivo* converted DNT significantly limited ConA-induced liver injury. Serum aminotransferase (ALT) levels ((**c**), *P<*0.001, *n=*4 in each group) were significantly decreased compared with the ConA group. The severity of hepatocyte necrosis, shown by pathological analysis, was also significantly reduced in the pretreated-*ex vivo* converted DNT group (**d**). Paraffin sections, original magnification: × 200. **P<0.01, *P<0.05

In this study, ConA-induced DNT expressed the perforin gene at levels significantly higher than those in CD4^+^ T cells (Figure 1e). To test the role of perforin in ConA-induced DNT-mediated suppression, we compared the suppressive function of DNT induced from WT C57BL/6 mice with that of cells from perforin-KO C57BL/6 mice *in vivo*. We injected perforin-KO mice and WT mice with ConA, then isolated DNT from both sets of mice 2 days later. Next, we adoptively transferred the isolated cells into naïve WT mice, and then administered ConA. The results of this assay are shown in Figures 6a and b. The mice that received WT DNT before the ConA challenge showed significantly lower levels of serum ALT and no obvious hepatocyte necrosis. However, the protective effect was partially abrogated by knocking out perforin expression in DNT. The mice that received perforin-KO DNT before ConA challenge showed significantly higher serum ALT levels and significant hepatocyte necrosis. These results suggested that perforin played an important role in DNT-mediated regulation.

### *Ex vivo* converted DNT from CD4^+^ T cells protect the liver from immune-mediated injury

To test the potential application of utilizing this novel differentiation pathway in immune-mediated liver damage, we isolated CD4^+^ T cells from WT C57BL/6 mice, and converted CD4^+^ T cells into DNT *in vitro* with ConA challenge, then adoptive transferred these *ex vivo* converted DNT to C57BL/6 mice followed by ConA administration. As shown in Figures 6c and d, *ex vivo* converted DNT significantly limited ConA-induced liver injury. Serum ALT levels were significantly decreased compared with the ConA group. The severity of hepatocyte necrosis, shown by pathological analysis, was also significantly reduced in the *ex vivo* converted DNT-pretreated group. These results suggested that *ex vivo* converted DNT also served as an immune regulator in the protection of ConA-mediated liver injury, and may have the potential feasibility of utilizing this novel differentiation pathway for the prevention and treatment of immune-mediated liver injury.

## Discussion

The immune system has evolved to exert an effective defense against pathogens as well as to minimize excessive immune-mediated tissue damage caused by immune responses against self-, allo- and environmental antigens. ConA-induced liver injury in mice is a well-characterized model of T cell-mediated liver disease depends on the activation of T lymphocytes by macrophages in the presence of ConA and has been extensively used to mimic many aspects of human T cell-mediated liver disease.^12, 21, 22^ During immune-mediated liver injury, how immune system appropriately downregulates activated immune components and restores its balance still needs to be further investigated.

DNT compose only 1–5% of total T cells in mice and humans, and have strong suppressive activity toward CD4^+^ T cells and CD8^+^ T cells,^11, 23, 24, 25^ as well as B cells,^23, 26^ dentritic cells^27^ and NK cells.^28^ In this study, in addition to the expected pathological necrosis of hepatocytes described after ConA treatment, we found that peripheral DNT were unexpectedly induced, increasing up to threefold in a dose- and time-dependent manner compared with untreated controls. We also provide key evidence that these ConA-induced DNT are regulatory cells that protect mice from immune-mediate liver injury through inhibition on the activation of lymphocytes and proinflammatory cytokine secretion. Fluorescence-activated cell sorting analysis confirmed that ConA-induced DNT were TCRβ^+^, B220^+^, CD44^+^ and CXCR3^+^. These DNT did not express Foxp3, and they expressed very low levels of IL-2, IL-4, IL-10 and IL-17a, but high levels of IFN-γ and granzyme B. Perforin, a cytotoxic-related gene, was expressed by DNT at an extremely high level. When mice received perforin-KO DNT before ConA challenge, the protection of DNT against this immune-mediated liver injury was significantly reduced, suggested that perforin plays an important role in DNT-mediated immune regulation. Furthermore, using an adoptive transfer model and *in vitro* migration assay, we found that DNT home to the liver and its draining lymph node to exert their protective function. In addition, the interaction between CXCR3 and its ligands CXCL9/CXCL10 plays an important role in DNT migration.

More importantly, our study provided convincing evidence that the origin of these induced DNT was CD4^+^ T cells rather than CD8^+^ T cells. ConA-induced CD4^+^ T-cell proliferation is APC dependent, and one of the major co-stimulatory complexes, OX40–OX40L, was demonstrated in this study to be involved in the process of CD4^+^ T-cell conversion to DNT. OX40 has been shown to be important in positive regulation of the anti-apoptotic molecules Bcl-XL, BCL2 and IAP, which results in increased T-cell survival.^29, 30, 31^ In mature T cells, programmed cell death following activation is considered a mechanism for termination of an immune response and for the maintenance of peripheral tolerance. Thus, proliferating CD4^+^ T cells may convert to regulatory DNT to maintain peripheral immune tolerance and minimize immune-mediated tissue damage when T-cell apoptosis is impaired. This process could explain the phenomenon where a higher proportion of conversion to DNT has been found in WT CD4^+^ T cells than in OX40-KO CD4^+^ T cells. To further confirm this hypothesis, and because the Fas/FasL interaction is another important modulator in T-cell apoptosis, CD4^+^ T cells were purified from B6.*lpr* mice (Fas deficient) and co-cultured with splenocytes under ConA stimulation. As expected, a higher proportion of *lpr*-CD4^+^ T cells than WT CD4^+^ T cells converted into DNT (data not shown). Massive accumulation of DNT has been observed in *lpr* mice, and the origin and function of *lpr* DNT have been widely debated.^32, 33, 34, 35^ Based on our results here, we suggest that at least some of the accumulated DNT in *lpr* mice might be converted from activated CD4^+^ T cells because of immune system losses of Fas-mediated apoptosis and that they could serve as an immune regulator in this lupus model. However, how the immune system initiates the conversion of CD4^+^ T cells to DNT cells remains unclear. Future studies are clearly needed to determine the underlying molecular mechanisms that mediate the pathways of CD4^+^ T-cell conversion to DNT.

Accumulating evidence has revealed that CD4^+^ T cells can acquire regulatory functions and differentiate to Foxp3^+^ or IL-10-producing type 1 regulatory cells to maintain immune homeostasis.^36, 37, 38, 39^ Scattered reports including ours described that CD4^+^ T cells can also convert into DNT *in vitro* under specific culture conditions.^11, 40^ However, this differentiation pathway from CD4^+^ T cells to DNT *in vitro* had yet to be confirmed as a truly intrinsic mechanism for maintaining immune system homeostasis in physiologic or pathologic processes. Our work provides key evidence that this novel differentiation pathway from CD4^+^ T cells to DNT *in vivo* shifts the balance from a destructive T-cell response to one that favors regulation. Therefore, in addition to apoptosis or conversion to Foxp3^+^ Treg or Tr1 cells, there is another important pathway for CD4^+^ T cells to convert from immune invader to defender, that is, DNT regulatory cells. This process allows these cells to counteract hyper-activated immune systems to ultimately restore homeostasis.

In conclusion, we have described for the first time a novel differentiation pathway where CD4^+^ T cells become regulatory DNT and exert significant protection against immune-mediated liver injury. Our study has revealed an intrinsic mechanism of maintaining immune system homeostasis *in vivo*, and provides key evidence that CD4^+^ T cells that convert into DNT could be an important therapeutic target for the prevention of autoimmune diseases, and supported the potential feasibility of utilizing *ex vivo* converted DNT from CD4^+^ T cell.

## Materials and Methods

### Mice

Male WT C57BL/6, C57BL/6 GFP^+^, C57BL/6 congenic for CD45.1, C57BL/6 perforin-Knock out (KO) and C57BL/6 OX40-KO mice were purchased from Jackson Laboratories (Bar Harbor, ME, USA). The mice were maintained in a pathogen-free environment in the animal facilities at the Beijing Friendship Hospital and all protocols were approved by the Institutional Animal Care and Ethics Committee.

### Reagent and antibodies

ConA (type IV) was purchased from Sigma (St. Louis, MO, USA). Mouse T-cell enrichment columns and neutralizing antibodies against B7.1, B7.2, OX40L and CD40L were obtained from R&D Systems (Minneapolis, MN, USA). Purified antibodies against mouse CD3, CD28 and cytometric Bead Array for mouse IFN-γ were purchased from BD Biosciences. Fluorochrome-conjugated antibodies against mouse CD3, CD4, CD8, CD19, CD44, CD45.1, CD45.2, B220, NK1.1, TCRαβ and CXCR3 were obtained from eBioscience (San Diego, CA, USA). ALT Detection Kits were purchased from NanJing JianCheng Biochemical Institute (Nan Jing, Jiang Su, China).

### Immune-mediated liver injury induction

To induce immune-mediated liver injury, mice received intravenous injection with 10 mg/kg ConA. Serum aminotransferase (ALT) and IFN-γ levels were evaluated 24 h after ConA injection according to the manufacturer's instructions. Liver samples were routinely fixed in 10% formalin and embedded in paraffin, sectioned into 5-μm slices and stained with hematoxylin-eosin.

### *In vitro* DNT conversion test

CD3^+^CD4^+^ or CD3^+^CD8^+^ T cells isolated from WT C57BL/6 mice by cell sorting (purity >98%, 2 × 10^5^ per well) and labeled with Carboxyfluorescein diacetate succinimidyl ester (Molecular Probes, Eugene, OR, USA), were cultured with splenocytes (2 × 10^5^/well) from CD45.1 C57BL/6 mice in 96-well round-bottom plates, then treated with 5 μg/ml ConA. And, 72 h later, CD45.2-positive cells were analyzed by flow cytometry.

### DNT isolation and adoptive transfer

DNT (1 × 10^6^, purity >97%) were sorted from splenocytes of WT C57BL/6 or perforin-KO mice 48 h after ConA administration, and transferred to naïve C57BL/6 mice by tail vein injection. *Ex vivo* converted DNT sorted from *in vitro* culture also transferred to naïve C57BL/6 mice. On same day, 10 mg/kg ConA was administered. Twenty-four hours after the injection, serum and liver samples were collected for ALT assays and histopathology, respectively.

### *In vitro* DNT suppression assay

CD3^+^ T cells (5 × 10^5^ cells/well) enriched by T-cell enrichment columns from splenocytes of CD45.1 C57BL/6 mice were treated with 5 μg/ml anti-CD3 and 2 μg/ml anti-CD28 as effector cells, and cultured with or without ConA-induced DNT or naïve DNT at a ratio of 2:1 in 96-well flat-bottom culture plates. After 3 days, EdU (RiBoBio, Guangzhou, China) were added to the plates 12 h before harvesting (final concentrations is 50 μM). EdU incorporation and Annexin V staining of CD45.1 positive T cells was determined by flow cytometry.

### *In vitro* cytotoxic assays

Single-cell suspensions from the liver were isolated using a GentleMACS according to the manufacturer's instructions (Miltinyi Biotec, Bergisch Gladbach, Germany). Hepatocytes were enriched by low-speed centrifugation at 50 × *g* for 5 min and used as target cells. Hepatocytes (1 × 10^5^/ml) from WT C57BL/6 were cultured in 96-well flat-bottom plate with or without splenocytes (2 × 10^5^/ml, from CD45.1 C57BL/6 mice) or ConA-induced DNT (1 × 10^5^, from WT C57BL/6) under 5 μg/ml ConA stimulation *in vitro*. Four hours later, hepatocytes were harvested and stained with Annexin V.

### *In vitro* DNT migration assays

DNT (0.4 × 10^6^) from ConA-treated C57BL/6 GFP^+^ mice were placed in the top chamber of Transwells with 8-μm pores (Corning Inc., Acton, MA, USA). WT C57BL/6 splenocytes (2 × 10^6^/ml) stimulated by ConA (5 μg/ml) were placed in the lower wells. The lower chambers contained complete medium with 0, 2.5 or 5 μg/ml CXCR3-173 (a neutralizing mAb against mouse CXCR3, BioXCell, West Lebanon, NH, USA). DNT cultured alone in the upper chamber served as negative controls. Transwell plates were incubated at 37 °C with 5% CO_2_ for 6 h, after which, the upper chambers were removed. Then, GFP^+^ DNT that had migrated to the lower wells were counted. Migration was quantified using inverted fluorescence microscopy by analyzing at least 10 random fields from each of three replicate filters for each experimental condition.

### Real-time PCR

Total RNA from cells or tissue samples was extracted using an RNeasy mini-kit (Qiagen, Valencia, CA, USA), and reverse transcribed to cDNA using SuperScript III RT-kit (Invitrogen, Carlsbad, CA, USA). Specific message levels were quantified by real-time PCR using the ABI 7500 Sequence Detection System (Applied Biosystems, Foster City, CA, USA). Primers and probes for mouse IL-2, IL-4, IL-10, IL-17a, FoxP3, FasL, IFN-γ, granzyme B and perforin were purchased from Applied Biosystems. Gene-specific primers used for CXCL9, CXCL10, CXCL11 were as follows: *CXCL9,* sense 5′-TGTGGAGTTCGAGGAACCCT-3′, antisense 5′-TGCCTCGGCTGGTGCTG-3′ *CXCL10,* sense 5′-ATCCCTGCGAGCCTATC-3′, antisense 5′-GCCATCCACTGGGTAAA-3′ and *CXCL11*, sense 5′-GCACCTCTTTCAGTCTGTTTCCTG-3′, antisense 5′-AGCCATCCCTACCATTCATTCAC-3′. The amplicon expression in each sample was normalized to *β-actin.* After normalization, gene expression was quantified using 2-ΔΔCt.

### Flow cytometry analysis

Cells from culture or recipient mice were harvested at various time points and analyzed for both proliferation and the expression of various cell surface markers. All samples were acquired on an Aria II flow cytometer (BD Biosciences). The data were analyzed using FlowJo software (Treestar, Ashland, OR, USA).

### Statistics

Statistical analysis was performed using the Prism 5.0 software (GraphPad Software, San Diego, CA, USA). The values are expressed as the mean±S.D. Analyses for significant differences were performed using Student's *t*-test and one-way ANOVA. *P*-values <0.05 were considered significant.

## Abbreviations

ALT: Aminotransferase
APC: Antigen-presenting cell
ConA: Concanavalin A
DNT: αβTCR^+^CD3^+^CD4^−^CD8^−^ double-negative T cells
EdU: 5-ethynyl-2′-deoxyuridine
FasL: Fas ligand
HBSS: Hanks' balanced salt solution
IL-2: interleukin 2
IL-4: interleukin 4
IL-10: interleukin 10
IL-17a: interleukin 17a
IFN-γ: interferon-γ
KO: knockout
mAb: monoclonal antibody
NKT: natural killer T cells
Tregs: T regulatory cells
WT: wild type
