# Supplementary figures and images for: A novel differentiation pathway from CD4+ T cells to CD4− T cells for maintaining immune system homeostasis
Source: Cell Death Dis. 2016 Apr 14;7(4):e2193–. doi: 10.1038/cddis.2016.83 (PMC4855662; doi:10.1038/cddis.2016.83)

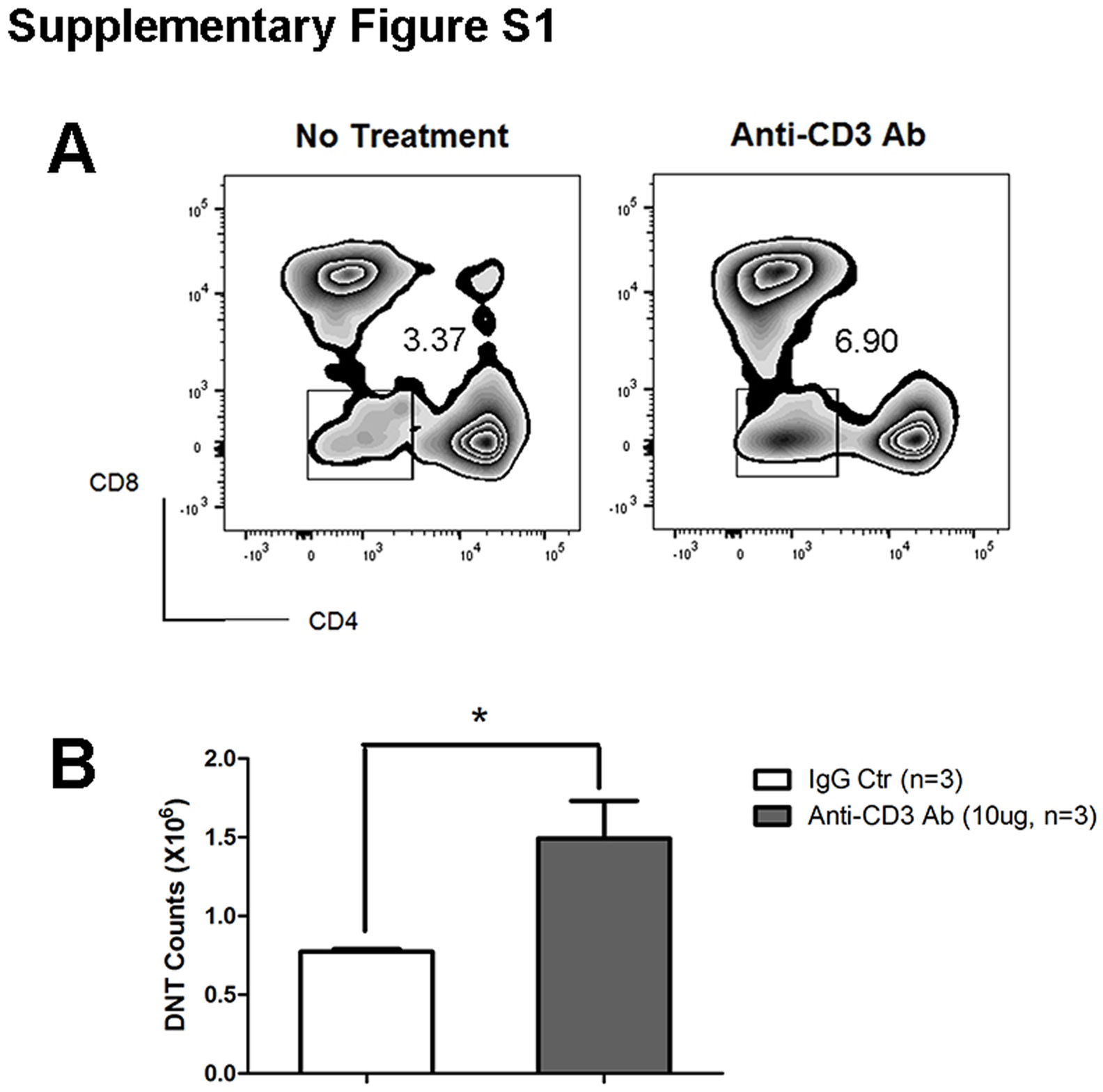

Supplement: Supplementary Figure 1 [file cddis201683x1.tif]

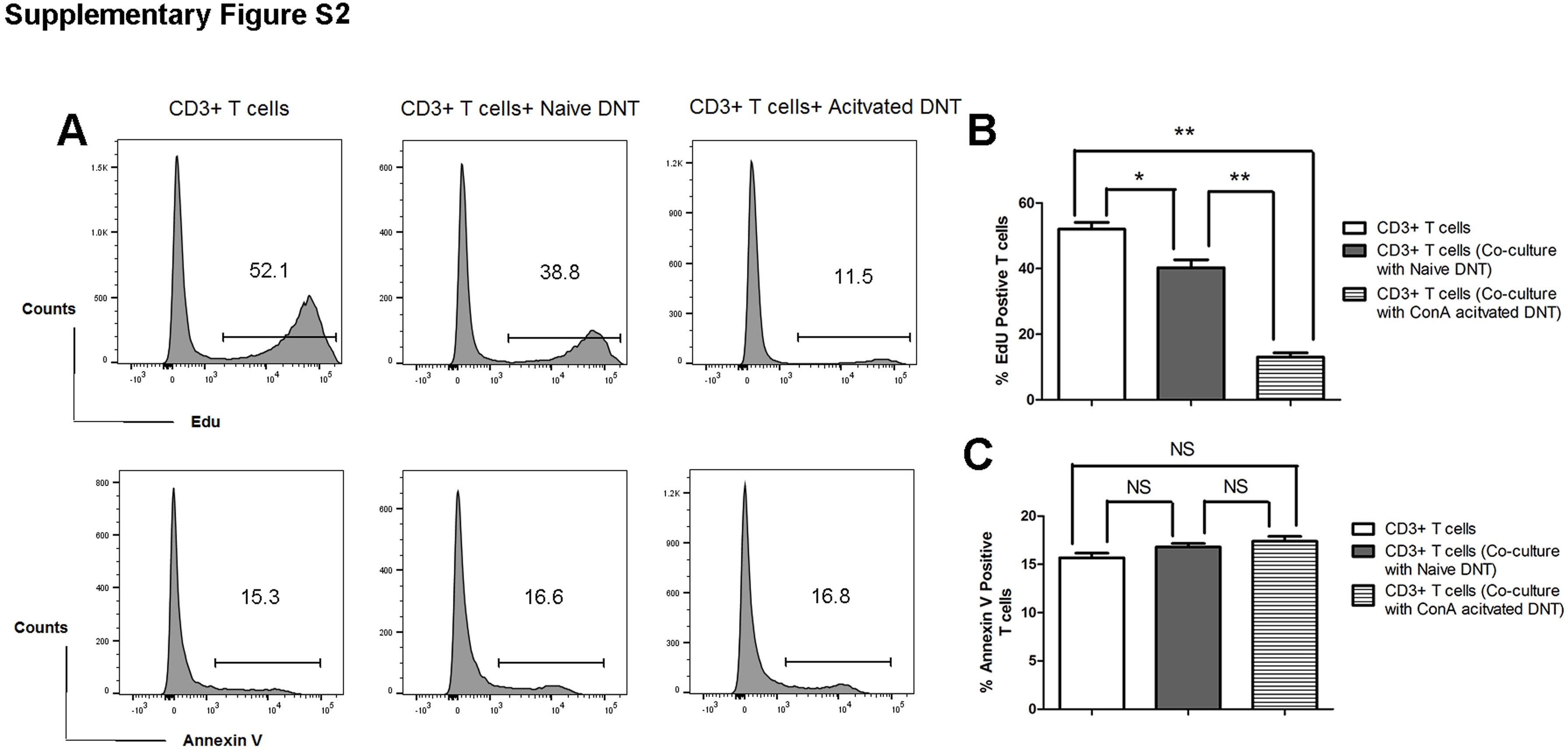

Supplement: Supplementary Figure 2 [file cddis201683x2.tif]
