# Supplementary material for: A novel differentiation pathway from CD4+ T cells to CD4− T cells for maintaining immune system homeostasis
Source: Cell Death Dis. 2016 Apr 14;7(4):e2193–. doi: 10.1038/cddis.2016.83 (PMC4855662; doi:10.1038/cddis.2016.83)
Supplement: Supplementary Figure Legends [file cddis201683x3.doc]

**Supplementary Figure Legends**

**Supplementary FigureS1. The proportion of CD3^+^CD4^-^CD8^-^ double-negative T cells (DNT) was up-regulated following anti-CD3 Ab administration in C57BL/6 mice.** DNT were significantly induced by anti-CD3 antibody administration, the percentage (**1A**) and absolute numbers (**1B**) of DNT in spleens were both increased significantly 48 hours after anti-CD3 antibody injection (n=3 in each group) (***p*<0.01, **p*<0.05, NS=No Significant Difference).

**Supplementary FigureS2. ConA-induced DNT had more profound suppression on T cell proliferation than naïve DNT.** Suppressive function of naïve or ConA induced DNT was assessed by EdU incorporation assay for proliferation **(2A, 2C) and** Annexin V staining for apoptosis (2B, 2C). Naïve CD3^+^ T cells from CD45.1 C57BL/6 mice were cultured with or without DNT in the presence of anti-CD3 (5ug/ml) and anti-CD28 (2ug/ml) for 72 h. Edu was added to the wells for 18 h before harvest. Results are expressed as mean±S.D. of triplicate cultures and are representative of three experiments with similar results. (***p*<0.01, **p*<0.05)
